# Temporal Design Patterns for Digital Phenotype Cohort Selection in Critical Care: Systematic Literature Assessment and Qualitative Synthesis

**DOI:** 10.2196/medinform.6924

**Published:** 2020-11-24

**Authors:** Daniel Capurro, Mario Barbe, Claudio Daza, Josefa Santa Maria, Javier Trincado

**Affiliations:** 1 School of Computing and Information Systems Centre for Digital Transformation of Health University of Melbourne Melbourne Australia; 2 Department of Internal Medicine School of Medicine Pontificia Universidad Catolica de Chile Santiago Chile; 3 Department of Biomedical Informatics Clinica Alemana Santiago Chile; 4 Instituto de Ciencias e Innovación en Medicina Facultad de Medicina Clínica Alemana, Universidad del Desarrollo Santiago Chile

**Keywords:** digital phenotyping, clinical data, temporal abstraction

## Abstract

**Background:**

Inclusion criteria for observational studies frequently contain temporal entities and relations. The use of digital phenotypes to create cohorts in electronic health record–based observational studies requires rich functionality to capture these temporal entities and relations. However, such functionality is not usually available or requires complex database queries and specialized expertise to build them.

**Objective:**

The purpose of this study is to systematically assess observational studies reported in critical care literature to capture design requirements and functionalities for a graphical temporal abstraction-based digital phenotyping tool.

**Methods:**

We iteratively extracted attributes describing patients, interventions, and clinical outcomes. We qualitatively synthesized studies, identifying all temporal and nontemporal entities and relations.

**Results:**

We extracted data from 28 primary studies and 367 temporal and nontemporal entities. We generated a synthesis of entities, relations, and design patterns.

**Conclusions:**

We report on the observed types of clinical temporal entities and their relations as well as design requirements for a temporal abstraction-based digital phenotyping system. The results can be used to inform the development of such a system.

## Introduction

The increasing costs of health care [1] and the rapid advance of new discoveries create the need for streamlining the identification of effective health interventions. The evidence-based clinical practice paradigm promotes the generation of such knowledge through high-quality randomized controlled trials and systematic reviews [2]. However, when we consider the amount of resources required to conduct a randomized controlled trial [3], alternative ways to assess the effectiveness of clinical interventions become attractive.

The broad adoption of electronic health records (EHRs) [4] allows researchers to analyze routinely collected electronic clinical data to conduct comparative effectiveness research. A health care system that systematically analyzes clinical data to generate and test hypotheses should be able to learn from itself, becoming a learning health care system [5]. However, converting a traditional health care system into a learning one faces several organizational, societal, and data-related barriers.

“Good data” is a relative concept [6], because it depends on who the user is and what the data are being used for. When EHR data are collected primarily for direct patient care and not with the explicit objective of generating knowledge, a majority of the captured information is stored as free text or other types of unstructured format, limiting its reuse potential. Our group has estimated that 75% of all data elements required for calculating clinical quality measures are not available as structured and computable database fields [7]. Similar results have been found about the clinical information required for clinical trial or cohort eligibility criteria [8]. The combination of data and rules to specify the latter are denominated a phenotyping algorithm [9]; digital phenotypes are the cornerstone of generating new knowledge from routinely collected clinical data and of a learning health care system.

The value of structured data lies in its capacity of being computed without major processing, therefore several attempts have been made to overcome the lack of structured clinical data. A review by Shivade et al [10] reported that the most frequently used methods to automatically identify patient cohorts based on EHR phenotypes were rule-based systems, natural language processing, and machine learning techniques. In this review a majority of studies involved the use of diagnostic codes to select eligible patients. However, although wide variations are seen, diagnostic codes frequently present poor sensitivity and specificity to accurately determine patients’ conditions [11].

Despite current advances in the area, cohort building systems require a significant amount of effort to develop and test and, in real scenarios, the most commonly used strategy to deal with limited EHR data quality is to use a combination of simple rules and manual verification of clinical data from patient records [12]. Thus, the field is still open to new and complementary approximations to identify patient cohorts based on digital phenotypes.

Clinical researchers face many barriers when querying clinical databases to find patients that match a specific cohort definition. One problem is that querying clinical databases is a complex task requiring multiple interactions between clinical researchers and database experts. Among those complexities, inclusion criteria frequently define temporal patterns of clinical events, which need convoluted temporal database queries [13]. This is needed in up to 40% of studies [8]. Finding patients that meet certain temporal patterns of clinical events could be both a barrier—when systems that do not easily support this feature are not available—and a very powerful tool to accurately retrieve patient cohorts based on these temporal digital phenotypes. However, systems that easily support this feature are not readily available.

In this study, we systematically reviewed the critical care literature to characterize the temporal representation of inclusion criteria, interventions, and outcomes, used by clinical researchers when designing a clinical study. The product of this review is a set of basic temporal entities, temporal relations, and the resulting temporal phenotype design patterns. The results can be used to inform the design of temporal abstraction-based digital phenotyping systems.

## Methods

### Data Source

We conducted a systematic literature review of published articles in the critical care domain. Using the Web of Science Journal Citation Reports, we selected the top 5 critical care journals according to their impact factor. Paired reviewers (MB, CD, JT, and JS) manually reviewed all publications and decided on inclusion or exclusion according to criteria described in the following section. Disagreements were solved by consensus.

### Types of Studies Included

We included retrospective studies conducted in intensive care unit settings which used data obtained from EHRs, clinical databases generated from EHRs, or through manual chart abstractions. We excluded studies which presented exclusively outpatient or emergency department data.

### Data Extraction

**Figure 1:**
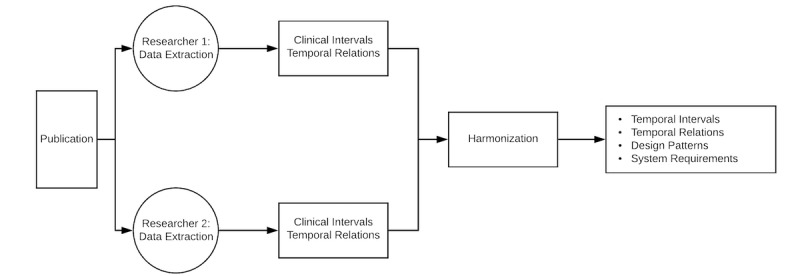
Overview of the data extraction process.

For every included study paired reviewers (MB, CD, JT, and JS) manually identified and extracted—using a purposefully built online form—all elements characterizing the study’s inclusion criteria, the interventions or exposures being studied (or the comparison group), and primary outcomes as defined by the original study authors following the Patient/Population, Intervention, Comparison, Outcome (PICO) framework [14]. Each attribute was then classified according to the clinical type (diagnosis, vital sign, laboratory result, medication, etc). When these elements contained a temporal dimension as defined by Boland et al [15], they were abstracted as *temporal intervals* or *instants*. For example, if the study included patients that underwent mechanical ventilation, because mechanical ventilation occurs during a period of time, such inclusion criteria would be abstracted as a mechanical ventilation interval; in the case of a single dose of antibiotics, that would be abstracted as a drug administration instant. Attributes that were not suitable to be represented as temporal attributes—such as sex, race—were represented as nontemporal patient attributes. A representation of the data extraction process can be seen in Figure 1.

When possible, if an interval or instant was itself an abstraction of lower-level concepts, it was decomposed into its parts according to the description explicitly provided or cited by the authors. If there were no details in the paper, we used standard definitions, when available. For example, when a systemic inflammatory response syndrome [16] was used as an inclusion criterion, we abstracted its components as determined by systemic inflammatory response syndrome definition at the time of the study: body temperature, heart rate, respiratory rate, arterial CO_2_ pressure, and white blood cells. If standard definitions were not available, we did not decompose that interval and it was extracted as the authors described it. Clinical events that are stored as free-text format—whether because they are traditionally stored in this form or it is the only available format, such as radiology reports or surgical protocols—were not represented in the abstractions.

To minimize variability in the data extraction process, all researchers followed an initial training period. Researchers classified the identified elements—inclusion criteria, interventions or exposures, and outcomes—using the framework described above. Discrepancies on the concept extraction and temporal representations were resolved by group agreement. We performed descriptive statistics from the concept extractions and the temporal elements obtained.

The abstraction process was conducted iteratively and continued until the point of saturation. We predefined saturation as being met when including additional studies did not add any new types of temporal elements.

Finally, researchers systematically documented temporal and nontemporal relationships between the identified temporal elements. This allowed us to identify the temporal query design patterns present in the literature. Finally, we documented the required functionality for a novel temporal abstraction-based system to identify patient cohorts, interventions/exposures, and outcomes in large clinical databases.

## Results

### Data Extraction

After iteratively extracting clinical concepts, the point of saturation—where no new types of temporal elements were identified—was reached after reviewing 28 primary studies. We obtained a total of 362 clinical entities, 48.6% (n=176) were inclusion criteria, 24.3% (n=88) were classified as interventions or exposures, and 27.0% (n=98) were outcomes. Abstracted entities were further classified into categories according to their clinical type, which are described, with examples, in Table 1. Therapeutic interventions (26.2%, 95/362), diagnostic tests (20.7%, 75/362), and vital signs (11.3%, 41/362) categories covered almost 60% of all entities.

**Table 1:** Categories, examples, and frequencies of identified clinical entities (N=362).

Classification	Example	Count, n (%)
Therapeutic intervention	Drugs or procedures: vancomycin, orotracheal intubation	95 (26.2)
Laboratory/diagnostic tests	Serum creatinine, hematocrit	75 (20.7)
Vital signs	Body temperature, respiratory rate, central venous pressure	41 (11.3)
Diagnosis	Pneumonia, urinary infection	35 (9.7)
Patient location	Intensive care unit hospitalization, patient transfer	26 (7.2)
Clinical scores	APACHE II^a^, Cerebral Performance Category	25 (6.9)
Nontemporal attribute	Sex, ethnicity	17 (4.7)
Death	In-hospital deaths, 30-day mortality	15 (4.1)
Physical examination finding	Pupil diameter, abdominal pain	11 (3.0)
Past medical history	History of trauma	7 (1.9)
Disposition	Discharge to home, institution, or other health center	5 (1.4)
Other	Appropriate antibiotic usage	10 (2.8)

^a^APACHE II: Acute Physiology And Chronic Health Evaluation II.

### Temporal Entities—Instants and Intervals

Of the 362 abstracted entities, 328 could be classified as clinical instants or intervals. Most entities could be abstracted as instants (54.1%, 196/362). This type of abstraction is used to represent a clinical event that does not have a duration but has a timestamp. For example, one inclusion criteria in this category was “the presence of arterial lactate > 2.5 mmol/L.” As much as 36.5% (132/362) of abstracted entities were of type interval. This type of abstraction is used to represent a clinical event that has a duration—defined by a start and end time—greater than 0. An example of a clinical interval is “noninvasive mechanical ventilation for at least 48 hours.”

### Types of Clinical Intervals

Further analysis of clinical intervals showed that they can also be subdivided into 3 different categories:

- Instant-based intervals: clinical intervals that are abstractions of identical instants. An example of this is hypothermia interval in which the interval is an abstraction of multiple instants of low body temperature measurements. Sometimes specific conditions have to be met to abstract this kind of interval: a time interval for a patient receiving more than 100 mL/hour of intravenous fluids. In other occasions, the instants were only used as categorical variables, regardless of the quantity: patient receiving normal saline infusion.
- Bounded intervals: clinical intervals that are abstractions of specific instants defining their start and end times. An example of this is a hospitalization interval, where the start is defined by an admission instant and the end is defined by a discharge instant. Additional arithmetic operations may need to be applied to these intervals, for example, a clinical interval describing a hospitalization longer than 7 days.
- Moving window intervals: clinical intervals where a specific condition needs to be met during a predefined window of time. An example of this was an oliguria interval, in which the condition oliguria (urinary output < 0.5 mL/kg/hour) has to be met during a 6-hour window. This denomination is consistent with previous descriptions [17].

**Figure 2:**
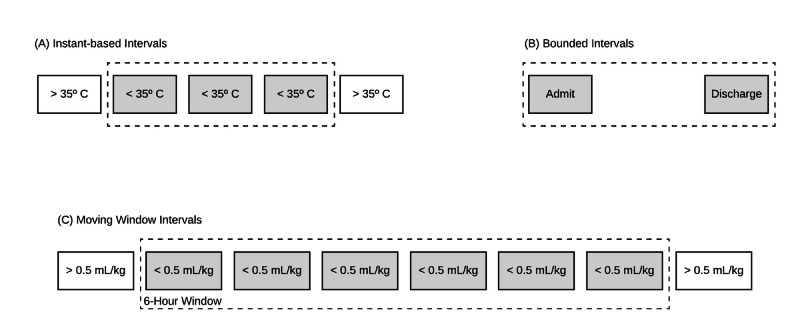
Three observed categories of clinical temporal intervals.

Graphic examples of the 3 types of intervals are presented in Figure 2.

### Within-Interval Calculations

In a small subset of intervals, arithmetic calculations were needed to correctly abstract them. For example, calculating an interval of pulse pressure variation (PPV) within a defined range would require calculating PPV (%) = 100 × 2 ([PP_max_ – PP_min_]/[PP_max_ + PP_min_]) at each instant before executing the abstraction. Other examples of within-interval calculations included counting the number of instants occurring inside an abstracted interval. An example of this would be an outcome defined as the number of chest x-rays performed on each patient during his or her stay in the intensive care unit; the interval is of type bounded (admission/discharge from the intensive care unit) and we need to count the number of additional instants (chest x-rays) occurring within the interval.

### Temporal and Atemporal Relations

**Figure 3:**
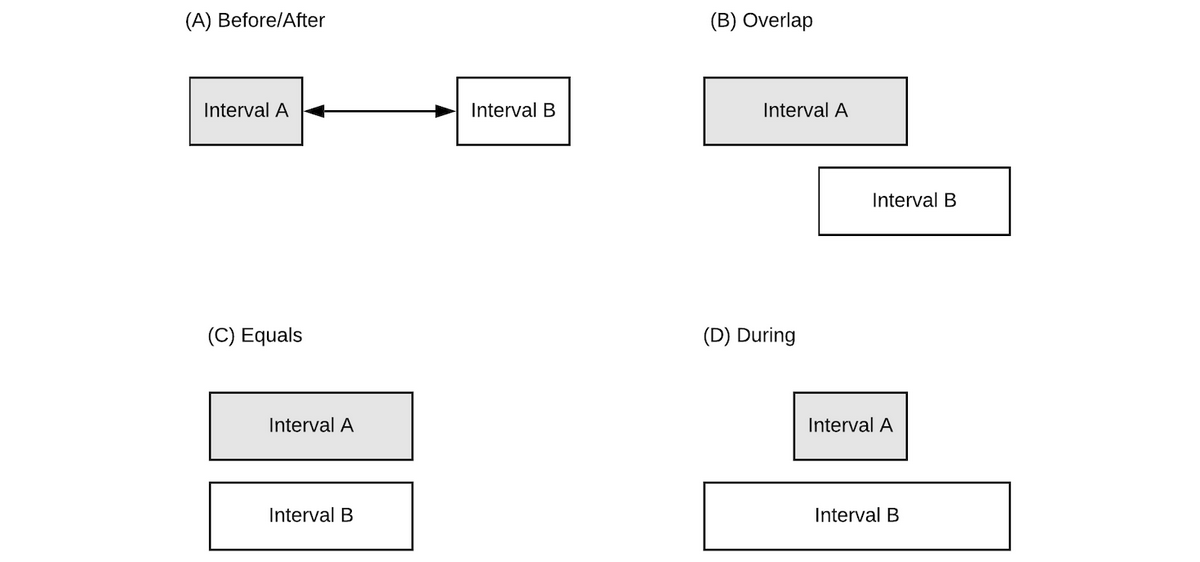
Examples of temporal relations.

We explored the temporal relations between instants and intervals and, as expected, all of them conformed to the temporal logic described by Allen [18]. Briefly, Allen described 13 possible temporal relations between a pair of intervals. Examples of these are the before, equal, and overlap temporal relations, among others. Graphic examples are presented in Figure 3.

In addition, some intervals were constructed by combinations of Boolean relations between intervals and instants. For example, to adequately represent a pediatric sepsis interval as defined by the International Pediatric Sepsis Consensus Conference [19] as required by the study authors, we required the Boolean relation AND between 6 different instants, and each one of them temporally related to an instant-based interval.

Some of the extracted entities did not have a temporal component and were denominated nontemporal patient attributes. Examples of these are age, race, and sex.

Finally, 5.5% (20/362) of the extracted concepts were not able to be represented using this proposed framework. For example, the outcome appropriate antimicrobial administration defined as whether the isolated bacteria were susceptible to the administered antibiotic implies a qualitative interpretation of a laboratory examination, which is out of scope of a temporal representation of clinical entities.

### Nested Queries

One additional functionality that was particularly salient was the need to perform nested queries. In a nested query, a query uses the output of another query as its input. Observational studies frequently explore the effect of a specific exposure; this study design involves creating 2 patient cohorts that are identical except for the exposure. When the outcome is assessed in both cohorts, a nested query is the most natural way to satisfy this requirement:

SELECT (Outcome Phenotype) FROM (Exposure Cohort)

In this case *Outcome Phenotype* and *Exposure Cohort* are both themselves queries.

### Design Patterns

**Figure 4:**
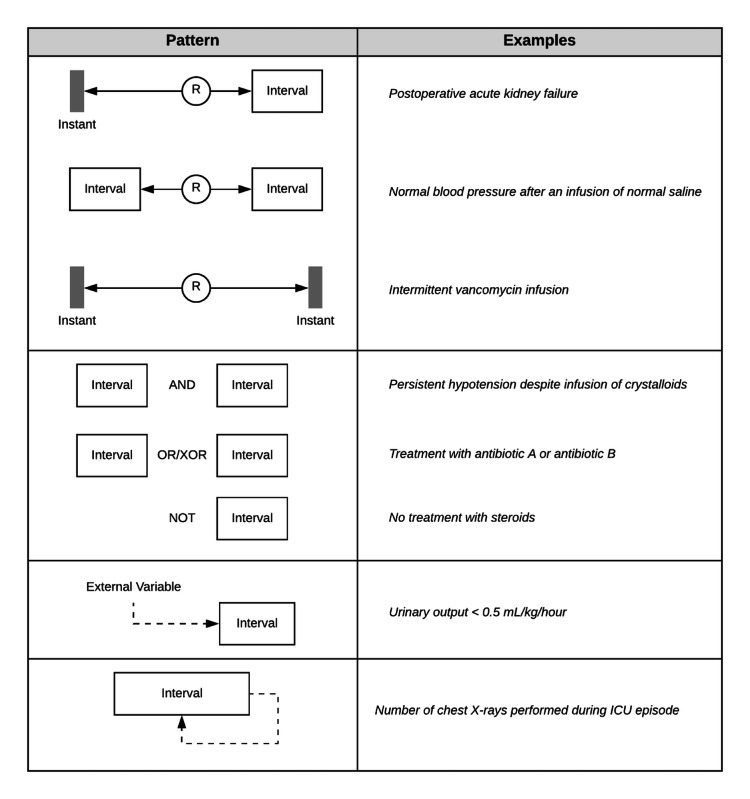
Examples of identified clinical temporal design patterns. ICU: intensive care unit.

The combination of temporal entities (instants and intervals), temporal relations, and nontemporal patient attributes can be used to describe the different observed patterns. A graphic description is presented in Figure 4.

Intervals can be temporally related to either instants or intervals. The same was observed for instants. We observed all 13 temporal relations described by Allen [18]. We call a pair of temporally related temporal entities (ie, Interval–Relation–Interval) a basic pattern. These basic patterns can, in turn, be related to other temporal entities or other temporal patterns. Those relations can be either temporal or through Boolean operators (AND, OR, Exclusive OR, NOT).

Intervals can use external variables as a condition to meet either before or after being abstracted. The first case would be the abstraction of an interval of reduced urinary output (<5 mL/kg/hour), in which each urinary output instant needs to be checked against the patient’s body weight (the external variable) before being added to the interval. An example for the second case could be *total dose of prednisone less than 10 mg/kg*. This interval is abstracted from individual instants of prednisone administration and after the interval is abstracted, it is checked against the patient’s body weight (the external variable). A final case was seen when an internal calculation—using information completely contained within the interval—was needed to be performed to generate the required attribute for the interval. An example of this would be an interval of a series of chest x-rays and, at the end, the number of x-rays would be calculated to create the interval total number of chest x-rays per week.

## Discussion

### Main Findings

This study presents a systematic, literature-based assessment of design requirements to develop a temporal abstraction-based digital phenotyping tool. Such a tool would facilitate the conduction of retrospective clinical studies in critical care using routinely collected electronic clinical data through enabling a rich description of clinical phenotypes. Once validated, these temporally abstracted digital phenotypes should be able to correctly represent patient cohorts, clinical interventions or exposures, as well as relevant clinical outcomes. The iterative nature of this review, which was conducted until reaching information saturation, adds robustness to its findings.

The initial findings of this review are consistent with previous research describing the nature of temporal clinical entities, in the form of clinical instants and intervals [20], as well as temporal relationships between these entities [18]. Other temporal abstraction-based digital phenotyping systems have been described in the past [21,22]; however, there are no reports that their development has been informed by systematically reviewing observational studies. As a consequence, this study adds 3 additional functionalities that may facilitate the creation of digital phenotypes for observational research.

First, this review shows that 3 subtypes of clinical intervals—instant-based, bounded, and moving window—are necessary to adequately represent digital phenotypes. Second, in addition to these interval subtypes, there is a need to perform calculations both within a clinical interval and with data external to the interval being abstracted. The third component involves the need to allow for nested queries when building digital phenotypes for observational studies. Other findings of this systematic review confirm the need to query for temporal relations and Boolean relations as described by Mo et al [23] in their desiderata for digital phenotyping. Finally, it is essential to highlight the need to generate high-quality temporal metadata during routine clinical documentations because temporal queries are an essential component of digital phenotyping.

### Limitations

The main limitation of this review is its focus only on intensive care studies. We chose this setting given the temporal density of clinical data collected during critical care episodes. We cannot claim that these findings will be similar in other clinical domains; that statement would need to be explicitly verified in additional studies. A second limitation is the exclusion of inclusion criteria based on free text contained in clinical notes or reports. This was an explicit decision given our goal of designing a digital phenotyping system able to abstract higher-level concepts from structured data without relying on free text. We still need to demonstrate the feasibility of this approach [24].

## Abbreviations

APACHE II: Acute Physiology And Chronic Health Evaluation II
EHR: electronic health record
PICO: Patient/Population, Intervention, Comparison, Outcome
PPV: pulse pressure variation
